# Assessing the Performance of a Novel Bayesian Algorithm at Point of Care for Red Eye Complaints

**DOI:** 10.3390/vision6040064

**Published:** 2022-10-24

**Authors:** Alexander M. Deans, Amy Basilious, Cindy M. Hutnik

**Affiliations:** 1 Schulich School of Medicine and Dentistry, Western University, 1151 Richmond St., London, ON N6A 5C1, Canada; 2 Department of Ophthalmology, Schulich School of Medicine and Dentistry, Western University, 1151 Richmond St., London, ON N6A 5C1, Canada

**Keywords:** algorithm, diagnosis, differential, red eye

## Abstract

The current diagnostic aids for red eye are static flowcharts that do not provide dynamic, stepwise workups. The diagnostic accuracy of a novel dynamic Bayesian algorithm for red eye was tested. Fifty-seven patients with red eye were evaluated by an emergency medicine physician who completed a questionnaire about symptoms/findings (without requiring extensive slit lamp findings). An ophthalmologist then attributed an independent “gold-standard diagnosis”. The algorithm used questionnaire data to suggest a differential diagnosis. The referrer’s diagnostic accuracy was 70.2%, while the algorithm’s accuracy was 68.4%, increasing to 75.4% with the algorithm’s top two diagnoses included and 80.7% with the top three included. In urgent cases of red eye (*n* = 26), the referrer diagnostic accuracy was 76.9%, while the algorithm’s top diagnosis was 73.1% accurate, increasing to 84.6% (top two included) and 88.5% (top three included). The algorithm’s sensitivity for urgent cases was 76.9% (95% CI: 56–91%) using its top diagnosis, with a specificity of 93.6% (95% CI: 79–99%). This novel algorithm provides dynamic workups using clinical symptoms, and may be used as an adjunct to clinical judgement for triaging the urgency of ocular causes of red eye.

## 1. Introduction

Patients often present to healthcare providers with a red eye and no clear diagnosis [1]. This leads to delayed management and extraneous referrals or tests [2,3].

Clinical specialists’ diagnostic ability stems from Bayesian thinking. Their differential diagnosis is continuously adjusted at each step of decision-making by history or physical exam findings (pre-test odds), which inform their next clinical step [4]. However, clinical generalists (non-ophthalmologists) who consult resources such as UpToDate and clinical practice guidelines will find no Bayesian diagnostic aids. UpToDate provides encyclopedic, accurate documentation on clinical diseases, but does not provide easy, stepwise approaches [5]. Clinical practice guidelines use static algorithms (traditional flowcharts) that provide general approaches but do not account for pre-test probability when proposing the next steps [6]. They necessitate the same series and number of questions to yield a diagnosis, rendering them inflexible and inefficient [7,8].

Machine learning and artificial intelligence (AI) are increasingly being integrated into clinical settings for diagnostic aid. Within ophthalmology, they are helping to automate the detection or tracking of corneal ulcers, corneal ectasia, and iritis and to predict the prognosis of various diseases [9]. Improving diagnostic accuracy at the patient’s initial contact with the health care system can improve triaging [10]. However, current AI tools are largely image-based and track quantitative findings, and do not consider clinical symptoms or signs.

A robust, dynamic algorithm that streamlines a medical workup may fill the gap in available diagnostic aids at point of care. The author (AMD) developed Pickle, a novel app that employs Bayesian algorithms, to provide primary care practitioners with workups for common ophthalmic complaints. The algorithms use a dynamic Bayesian feedback process to continuously recreate themselves at each decision-making step.

This study tested the app’s red eye algorithm by comparing its diagnostic accuracy to that of referring physicians and ophthalmologists. The paper does not aim to present a detailed analysis of the algorithmic design itself, as this lies outside the medical scope.

## 2. Materials and Methods

### 2.1. Pickle App Design and Development

**Figure 1:**
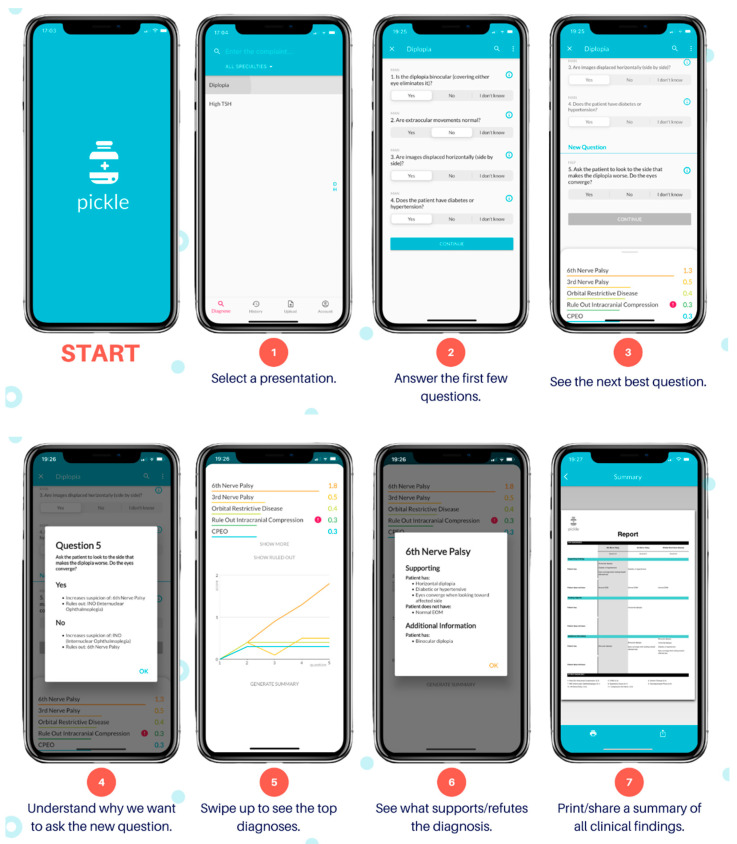
Pickle app user interface.

The author (AMD) developed Pickle, a novel mobile application with its user interface shown by Figure 1. Pickle first requires answers to 3–4 “Yes/No/I don’t know” questions to begin narrowing its differential diagnosis. It subsequently provides a differential diagnosis ranked by likelihood. Next, Pickle’s proprietary Bayesian-based artificial intelligence determines the most appropriate next workup step. Specifically, the differential diagnosis is parsed against the question bank to determine which questions can rule out diagnoses, significantly reduce their likelihood, or significantly increase their likelihood. Each question is stored with a table of values that represents its ability to stratify diseases by increasing or decreasing the level of suspicion depending on the answer. Employing Bayesian principles, a dynamic process ensues, whereby the question that garners the highest potential difference between pre-test and post-test probability is asked next to expedite decision-making. Further constraints are imposed on this dynamic order of questions, including the presence of prerequisite investigations and the elimination of questions that would add redundancy. For example, if the user answers “Yes” to “Is there corneal staining with fluorescein?”, the algorithm would prioritize the subsequent questions: “Is there contact lens use?” and “Is there a whitish corneal opacity?” to assess for a corneal ulcer. It would also eliminate the question “Is there chronic tearing?” from the question bank, as the answer would not add diagnostic value. Therefore, the algorithm’s novelty lies in its ability to adjust its order of questions to each case uniquely, as an ophthalmologist would, by balancing clinical suspicion with requisite investigations.

After each question, the algorithm re-sorts the differential by likelihood, displaying numerical and visual scores (Figure 1, Step 5) for each diagnosis that are indicative of its suspicion. The dynamic questioning is repeated until the user terminates the program or the level of suspicion for the top diagnosis heavily outweighs that of the others. Moreover, users can select a diagnosis within the differential, and this will be provided with the symptoms or history that support or refute it. The algorithm may also provide simple management steps depending on the responses provided, such as “Refer to Ophthalmology to rule out a corneal ulcer.”

### 2.2. Study Design and Sample

This prospective study tests whether a dynamic Bayesian algorithm can improve the diagnostic accuracy of red eye complaints when compared to that of primary care physicians, using ophthalmology residents’ diagnostic impression as a gold standard.

**Figure 2:**
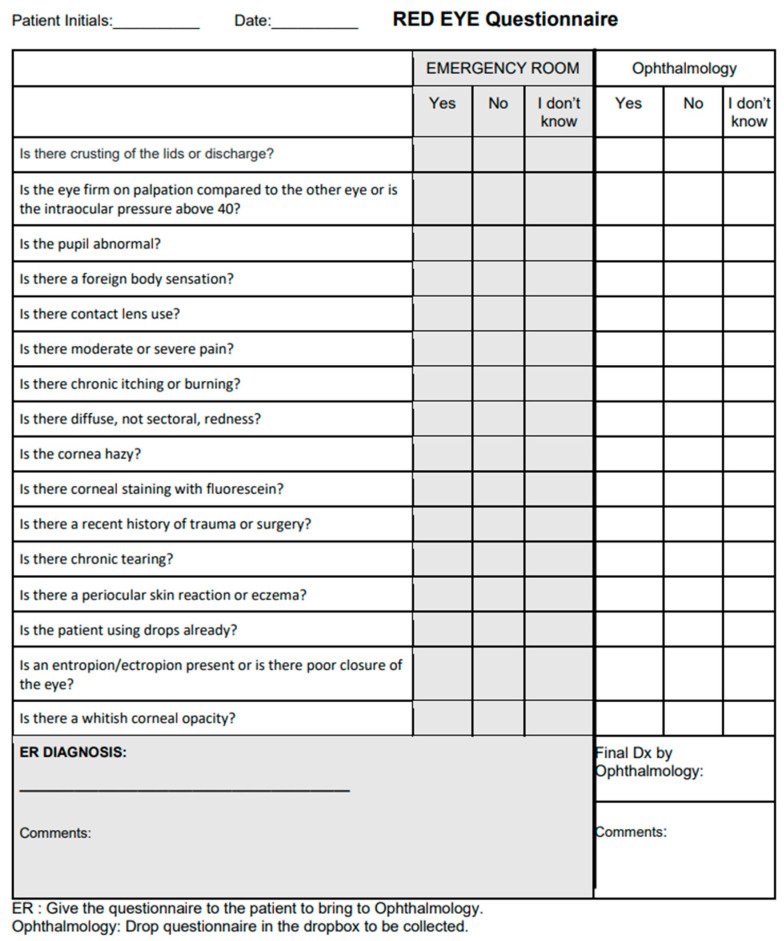
Questionnaire including all possible questions of the Pickle red eye algorithm.

A questionnaire was developed with a list of all possible algorithm questions (Figure 2) which prompted for medical history and physical examination findings. It did not necessitate extensive slit lamp findings, prompting only for the presence of large corneal opacities or areas of fluorescein uptake.

The paper questionnaire was distributed to emergency departments who referred adult patients with red eye to an ophthalmology urgent eye clinic in London, Ontario, Canada. Questionnaires prompted for the referring physician’s diagnosis and patients’ medical history and physical exam findings without using checkboxes or multiple-choice options. Patients who presented with simultaneous new vision loss or diplopia were included. The main inclusion criteria were (i) adults (18 years or older) who (ii) presented with red eye, as (iii) assessed by the referring physician with (iv) no prior diagnosis by ophthalmology. Patients were seen by the ophthalmology clinic less than one day after referral, with no clinical interventions in the interim. A consecutive series sample of 57 patients was completed. This sample size allowed for all common causes of red eye to be represented. Data were collected between March and August 2021.

Referrers completed the questionnaire with their suspected diagnosis (the “referrer diagnosis”), which was then sent with a referral to ophthalmology. One of eight resident ophthalmologists then assessed the patient and attributed a gold-standard diagnosis after reviewing with a staff ophthalmologist. Since the resident received the referral, he or she was not blinded to the referrer diagnosis. After the first 21 patients, the questionnaire was updated to also prompt the ophthalmology resident for his or her impression of the medical history and physical exam findings, as the research team aimed to correlate these—which sometimes differed from the referring doctor’s findings—to the ultimate diagnosis. Post-visit, questionnaire data were entered into Pickle’s red eye algorithm, generating an “algorithm differential”. If a response was not documented by the referrer, it was entered as “I don’t know” on the algorithm. The ophthalmology resident was blinded to the algorithm differential.

### 2.3. Algorithm Diagnoses

The algorithm may make 15 possible diagnoses for red eye:

1. Eczema or Reaction to Topical Medications;
2. Blocked Tearduct;
3. Lid Abnormality (ectropion, entropion, or lagophthalmos);
4. Blepharitis;
5. Dry Eye;
6. Subconjunctival Blood;
7. AVM;
8. Conjunctivitis;
9. Episcleritis;
10. Scleritis;
11. Keratitis/Corneal Abrasion;
12. Corneal Ulcer;
13. Acute Angle-Closure Glaucoma;
14. Iritis;
15. Endophthalmitis/Severe Inflammation.

This list of possible algorithm diagnoses was not available to the resident and staff ophthalmologist. However, it was available to the investigators who entered the clinical data from referrers into the algorithm to assess for diagnostic accuracy. Certain external pathologies that cause red eye, including cellulitis (pre-septal and orbital), hordeolum, chalazion, pingueculitis, blunt ocular trauma, and globe-penetrating injuries, were not included in the algorithm’s diagnostic options as they are usually clinically apparent.

A list of abbreviations and their meanings are shown in Table 1.

### 2.4. Outcome Measures and Data Analysis

The referrer diagnostic accuracy was calculated as the concordance between the “referrer diagnosis” and the “gold-standard diagnosis”. If no diagnosis was attempted by the referring physician, it was considered an incorrect diagnosis. Algorithm diagnostic accuracy was calculated as the concordance between the “algorithm differential” and the “gold-standard diagnosis”. Given that the algorithm ranks its differential diagnoses by likelihood, algorithm accuracy was calculated using three divisions: the accuracy of the top-scoring diagnosis (Top 1), the top two diagnoses (Top 2), and the top three diagnoses (Top 3).

The referrer and algorithm diagnostic accuracies were also computed for a subset of urgent cases. This permitted the calculation of sensitivity and specificity for the algorithm’s ability to identify cases as urgent or non-urgent.

## 3. Results

Questionnaires were completed for 57 patients referred with red eye between March and August 2021, with all included for analysis.

Based on the gold-standard diagnosis, the causes of red eye were: eczema or reaction to topical medications (*n* = 1), blocked tearduct (*n* = 0), lid abnormality (*n* = 0), blepharitis (*n* = 0), dry eye (*n* = 2), subconjunctival blood (*n* = 1), AVM (*n* = 0), conjunctivitis (*n* = 4), episcleritis (*n* = 0), scleritis (*n* = 4), keratitis/corneal abrasion (*n* = 16), corneal ulcer (*n* = 11), acute ACG (*n* = 1), iritis (*n* = 13), and endophthalmitis/severe inflammation (*n* = 1). Three cases were not able to be classified under the algorithm’s diagnostic options: post-operative pain (*n* = 1), and pingueculitis (*n* = 2).

### 3.1. Referrer Diagnostic Accuracy

**Figure 3:**
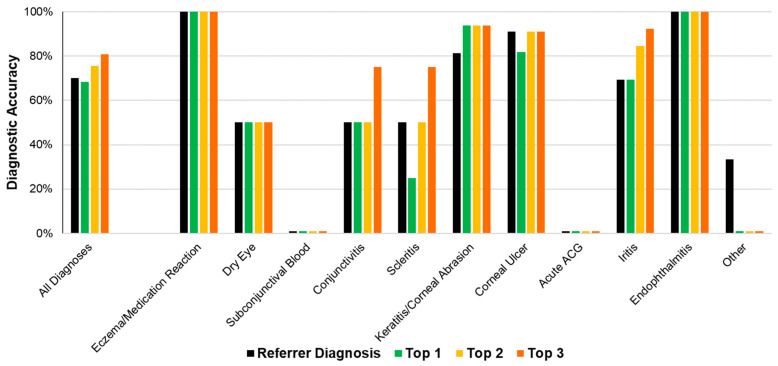
Referrer and algorithm diagnostic accuracies.

The referrer diagnosis was correct in 70.2% (40/57) of all cases (Figure 3). The most common referrer diagnoses were keratitis/corneal abrasion (*n* = 13), corneal ulcer (*n* = 10) ad iritis (*n* = 9). (Figure 3, Table 2).

### 3.2. Algorithm Diagnostic Accuracy

The algorithm’s diagnostic accuracy, using its top diagnosis, was 68.4% (39/57). When considering the top two diagnoses, accuracy was 75.4% (43/57). When considering the top three diagnoses, accuracy was 80.7% (46/57) (Figure 3). Table 2 displays the referrer and algorithm diagnostic accuracies for each independent diagnostic cluster.

### 3.3. Referrer and Algorithm Diagnostic Accuracy in Urgent Conditions

**Figure 4:**
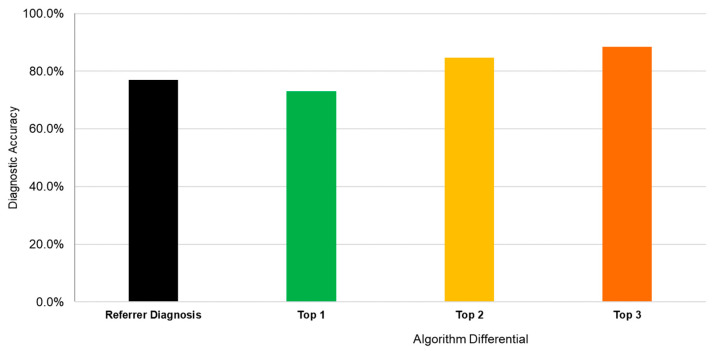
Referrer and algorithm diagnostic accuracy for urgent conditions.

Twenty-six cases were deemed “urgent conditions” requiring rapid referral to exclude serious pathology. These comprised diagnoses of corneal ulcer, acute ACG, iritis, and endophthalmitis/severe inflammation. The referrer diagnostic accuracy for urgent conditions was 76.9% (20/26) (Figure 4). The algorithm diagnostic accuracy for these urgent cases was 73.1% (19/26) when considering the top diagnosis. This increased to 84.6% (22/26) with the top two diagnoses and 88.5% (23/26) with the top three diagnoses.

For non-urgent cases, the algorithm was 64.5% accurate (20/31). The referrer sensitivity for determining that a case was urgent was 84.6% (95% CI: 65–96%), regardless of the diagnosis. The referrer specificity for the same was 83.9% (95% CI: 66–95%). However, the algorithm sensitivity for the urgency of a case was 76.9% (95% CI: 56–91%) using the top diagnosis, with a specificity of 93.6% (95% CI: 79–99%).

## 4. Discussion

Overall, the accuracy of the referring providers was 70.2% for all cases, increasing to 76.9% for urgent cases. Given that questionnaires were hand-written, anonymous, and non-prompted (no checkboxes for diagnoses), they were designed to encourage referrers to provide unbiased diagnoses. A recent study found that referrals to a regional emergency eye clinic were 45% accurate overall, with 54% for optometrists, 39% for emergency physicians and 33% for primary care practitioners [11]. This reveals that diagnostic decision aids would benefit non-ophthalmologists in assessing red-eye complaints.

Given equivalent clinical data, the algorithm overall improved on the referrer diagnostic accuracy (70.2%) to a range of 68.4–80.7%. A comprehensive slit lamp exam requires significant practice and skill to be executed well [12]. The Pickle algorithm, however, does not prompt referrers for more advanced findings such as anterior chamber cells, inquiring instead about gross, more apparent findings such as whitish corneal opacities or areas of focal fluorescein uptake.

The algorithm was designed to be highly specific to non-urgent conditions (subconjunctival blood, AVM, conjunctivitis, etc.) to impart confidence in referring when an urgent condition is proposed by the tool. The algorithm’s specificity in urgent cases was 93.6%, compared to a referral specificity of 83.9%. While the algorithm’s sensitivity for urgent cases was slightly lower (76.9%) than that of referrers (84.6%), this is primarily due to differences in clinical findings noted by referrers compared to the gold-standard clinicians. For example, several cases ultimately diagnosed with an urgent condition were noted by referrers to have absent ‘red flag’ signs or symptoms—such as the presence of a whitish corneal opacity—that were noted to be present when evaluated by ophthalmology. This is further discussed in the analysis of incorrect diagnoses below.

In eighteen cases, Pickle’s top diagnosis was not correct, with eleven of these not containing the gold-standard diagnosis in the top three differential diagnoses. The latter cases were analyzed to determine if the algorithm had behaved appropriately, given the presented clinical data. Of the eleven cases, the algorithm displayed desired judgement in seven cases, performed moderately well in three, and failed outright in one.

**Figure 5:**
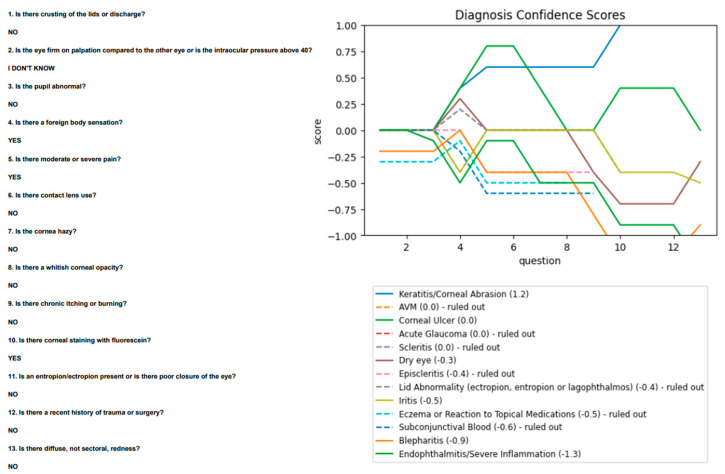
“Scleritis” case, using data from referrers.

First, a corneal ulcer (by gold-standard designation) was diagnosed by the algorithm as keratitis, a correct judgement given the presence of a whitish opacity in the absence of fluorescein uptake and foreign body sensation. However, it did textually prompt the user after the fourth question to rule out a corneal ulcer given the presence of an infiltrate. Second, a case of iritis was diagnosed as a corneal ulcer. The referrer noted a foreign body sensation, focal staining, and contact lens use, with ophthalmology later noting 0.5+ cells in the anterior chamber. These findings suggest a corneal ulcer rather than iritis, given the absence of moderate/severe pain or abnormal pupil. The cells are likely a secondary reaction. Third, conjunctivitis was diagnosed as keratitis. The patient presented with a foreign body sensation and diffuse redness without lid crusting or discharge, making conjunctivitis unlikely. Fourth, a subconjunctival bleed was diagnosed as keratitis. The referrer noted a history of trauma with moderate pain and fluorescein staining, attributing these to keratitis. The referring and algorithm diagnoses are appropriate, since a subconjunctival bleed does not cause corneal changes and is likely an accessory finding. Fifth, scleritis was diagnosed as keratitis. The referrer noted a foreign body sensation, moderate/severe pain, and corneal staining, leading to a diagnosis of keratitis appropriate (Figure 5). Scleritis does not usually present with a significant foreign body sensation.

**Figure 6:**
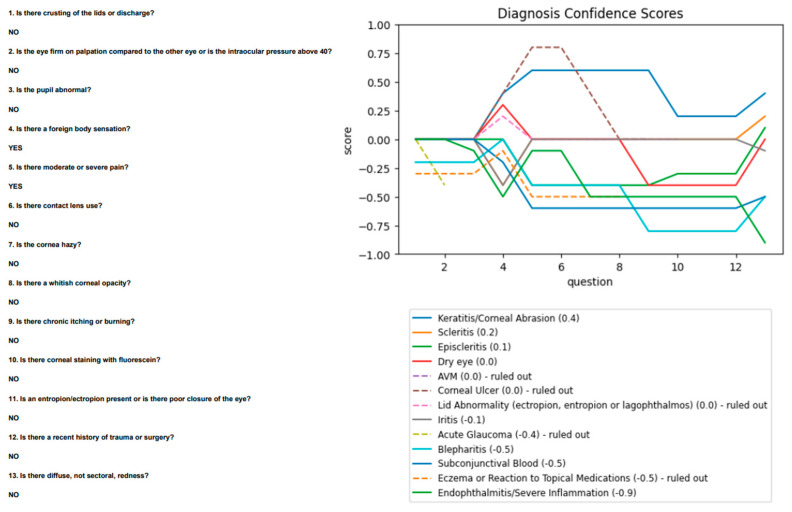
“Scleritis” case, using data from Ophthalmology.

Ophthalmology agreed with most findings but did not note staining. The algorithm was re-run with this change and proposed scleritis as its second diagnosis, after keratitis (Figure 6).

Sixth, a corneal abrasion was diagnosed as acute ACG. The referrer noted a firm eye with intraocular pressure greater than 40 mmHg and an abnormal pupil, consistent with acute ACG. Ophthalmology, however, noted normal pressures and a normal pupil, with which the algorithm was re-run and proposed a correct diagnosis of keratitis/corneal abrasion. Seventh, an acute ACG was diagnosed as iritis. The referrer noted an abnormal pupil, moderate/severe pain, and hazy cornea, with sectoral redness and normal pressures. This presentation is more consistent with iritis. Ophthalmology noted that the intraocular pressure increased from 26 mmHg in the emergency room to 55 mmHg at their assessment. When re-run, the algorithm’s top diagnosis was acute ACG. In these seven cases, therefore, the algorithm was effective at correlating the symptoms and history with a suitable diagnosis.

In two cases, pingueculitis was diagnosed by the algorithm as keratitis. Referrers noted corneal staining and moderate pain. Since pingueculitis was not included as a possible diagnostic option, keratitis is an appropriate candidate and should be ruled out. In another case, a dry eye was diagnosed as keratitis in a patient with lid crusting, foreign body sensation, and contact lens use. When assessed by ophthalmology, no crusting or foreign body sensation was noted, though the cornea did stain. This is consistent with keratitis, and it is unclear why a ‘dry eye’ diagnosis was attributed by ophthalmology. When re-run using the updated information, the algorithm proposed keratitis, dry eye, and a corneal ulcer as diagnoses. Pickle’s algorithm failed in one case of post-operative pain that it diagnosed as scleritis. The patient was only noted to have moderate pain. Given the absence of any positive symptoms or signs, the algorithm was unable to stratify diseases by their likelihood, and therefore provided an arbitrary, inappropriate diagnosis.

### Limitations

This study may be limited by the small sample size of patients presenting with eczema/reaction to topical medications, scleritis, and endophthalmitis. No patients with a blocked tearduct, lid abnormality, blepharitis, AVM, or episcleritis were assessed. This is likely due to the clinical setting of the trial. An emergency department has a selection bias compared to overall primary care settings, whereby serious and acute pathology such as iritis or corneal ulcers present more commonly. Additionally, only patients who were referred to specialist care were included, although they may be a representative sample of the target population since most patients presenting with an acute red eye are referred, as recommended by the American Academy of Ophthalmology and the Canadian Ophthalmological Society [13,14].

The algorithm was noted to be limited by user error. While Pickle failed to include the gold-standard diagnosis in its ‘top 3’ differential in eleven cases, it made appropriate judgements in at least seven given the history and physical exam findings presented to it. In particular, the presence and absence of corneal staining contributed to incorrect analysis, as the referrers and ophthalmology differed in their answer to this question in several cases. This suggests that further emphasis on history and symptoms rather than physical exam findings may aid primary care physicians and Pickle’s algorithms in making more accurate diagnoses.

This red eye algorithm helps to provide a practical approach to red eye. Its novelty lies in its dynamic nature: it adjusts the sequence of workup steps as it receives clinical findings, mimicking a specialist’s approach. Additionally, the algorithm provides a differential instead of a single diagnosis, better reflecting the nuances of clinical practice. Importantly, it ensures the continued consideration of critical diagnoses, irrespective of likelihood.

## 5. Conclusions

Primary care settings are often the first triage point for patients with red eye. This study obtained a referring diagnostic accuracy of 70.2%. Using Pickle’s Bayesian algorithms, diagnostic accuracy was improved to a range of 68.4–80.7%, although the research may be biased toward urgent and serious pathology, as it was conducted with patients presenting to an Emergency Department. Therefore, a small sample sizes was attained for benign pathologies. However, the algorithm’s high specificity in urgent cases (93.6%) makes it useful for decreasing the suspicion of acute ACG, iritis, corneal ulcers, and endophthalmitis. Furthermore, it provides this benefit using only the tools available to non-ophthalmologists, without requiring slit lamp findings other than noting white opacities and fluorescein uptake. It may, therefore, be used in primary care settings as an adjunct to clinical judgement to optimize referral decisions for serious and urgent conditions. By improving diagnostic accuracy and patient triaging, it may reduce resource utilization and improve patient care.

## Figures and Tables

**Table 1:** Abbreviations.

Abreviation	Meaning
AVM	Arterio-venous malformation
ACG	Angle-closure Glaucoma

**Table 2:** Referrer and algorithm diagnostic accuracies.

Gold Standard Diagnosis	Diagnostic Accuracy			
		Algorithm Differential		
	Referrer Diagnosis	Top Diagnosis	Top 2 Diagnoses	Top 3 Diagnoses
Eczema or Reaction to Topical Medications (*n* = 1)	1/1	1/1	1/1	1/1
Dry Eye (*n* = 2)	1/2	1/2	1/2	1/2
Subconjunctival Blood (*n* = 1)	0/1	0/1	0/1	0/1
Conjunctivitis (*n* = 4)	2/4	2/4	2/4	3/4
Scleritis (*n* = 4)	2/4	1/4	2/4	3/4
Keratitis/Corneal Abrasion (*n* = 16)	13/16	15/16	15/16	15/16
Corneal Ulcer (*n* = 11)	10/11	9/11	10/11	10/11
Acute ACG (*n* = 1)	0/1	0/1	0/1	0/1
Iritis (*n* = 13)	9/13	9/13	11/13	12/13
Endophthalmitis/Severe Inflammation (*n* = 1)	1/1	1/1	1/1	1/1
Other (*n* = 3)	1/3	0/3	0/3	0/3
Total	40/57	39/57	43/57	46/57

## Data Availability

The data presented in this study are available in the article.
